# Single-cell light-sheet fluorescence 3D images of tumour-stroma spheroid multicultures

**DOI:** 10.1038/s41597-025-04832-0

**Published:** 2025-03-24

**Authors:** Akos Diosdi, Filippo Piccinini, Timea Boroczky, Gabriella Dobra, Gastone Castellani, Krisztina Buzas, Peter Horvath, Maria Harmati

**Affiliations:** 1 Synthetic and Systems Biology Unit, HUN-REN Biological Research Centre, Szeged, Hungary; 2 Single-Cell Technologies Ltd, Szeged, Hungary; 3 Doctoral School of Biology, University of Szeged, Szeged, Hungary; 4 IRCCS Istituto Romagnolo per lo Studio dei Tumori (IRST) “Dino Amadori”, Meldola, (FC) Italy; 5 Department of Medical and Surgical Sciences (DIMEC), University of Bologna, Bologna, Italy; 6 Department of Immunology, University of Szeged, Szeged, Hungary; 7 Doctoral School of Interdisciplinary Medicine, University of Szeged, Szeged, Hungary; 8 IRCCS Azienda Ospedaliero-Universitaria di Bologna S.Orsola, Bologna, Italy; 9 Institute of AI for Health, Helmholtz Zentrum München, Neuherberg, Germany; 10 Institute for Molecular Medicine Finland, University of Helsinki, Helsinki, Finland

**Keywords:** Cancer models, Fluorescence imaging, Image processing, Cellular imaging

## Abstract

Spheroids are widely used in oncology for testing drugs, but models composed of a single cell line do not fully capture the complexity of the *in vivo* tumours targeted by chemotherapy. Developing 3D *in vitro* models that better mimic tumour architecture is a crucial step for the scientific community. To enable more reliable drug testing, we generated multiculture spheroids and analysed cell morphology and distribution over time. This dataset is the first publicly available single-cell light-sheet fluorescence microscopy image collection of 3D multiculture tumour models comprising of three different cell lines analysed at different time points. Specifically, we created models composed of one cancer cell line (melanoma, breast cancer, or osteosarcoma) alongside two stromal cell lines (fibroblasts and endothelial cells). Then, we acquired single-cell resolution light-sheet fluorescence 3D images of the spheroids to analyse spheroid morphology after 24, 48, and 96 hours. The image collection, whole spheroid annotations, and extracted features are publicly available for further research and can support the development of automated analysis models.

## Background & Summary

Three-dimensional (3D) cell cultures are of increasing interest in research as they provide an intermediate model system between 2D cultures and animal models^1^. They overcome the limitations of 2D cell cultures, such as (*i*) the lack of natural structures or cell-to-cell and cell-matrix interactions of native tissues, (*ii*) the unrestricted access to oxygen, nutrients, metabolites, and signal molecules, and (*iii*) altered cell morphology, polarity and functions, *e.g*., differentiation, proliferation, vitality, gene expression patterns and responsiveness to stimuli^2^. As 3D cultures closely resemble native tissues, they have become an essential model system in a wide range of research areas, including regenerative medicine^3^ and cancer research^4^.

In particular, the so-called multiculture 3D tumour spheroids mimic solid tumours in many aspects, such as the heterogeneous architecture, growth kinetics, physical interactions or complex communication^5^. Unlike 2D mono-cultures, they also represent the tumour microenvironment (TME), which is an active participant of all stages of cancer development^6,7^. However, today, even the classic 3D single-culture spheroids seem too simple for reliable high-content screening (HCS) analysis for predicting *in vivo* effects of chemotherapy treatments^8^. Current technology allows co-culturing tumour cells with healthy stromal cells, *e.g*., fibroblasts, mesenchymal stem cells, endothelial cells or immune cells, for obtaining advanced but reproducible preclinical tumour models for a wide variety of uses, ranging from basic research for the development of therapeutic approaches to advanced HCSs of chemotherapy drugs^9,10^. In this scenario, 3D multiculture spheroids seem to offer the current best trade-off for drug HCS, providing advantages over both classical 3D single-culture spheroids^6,7^ and the more complex yet less reproducible patient-derived tumour organoids^11^. While organoids possess self-renewal and self-organization capacities recapitulating the original tumour characteristics, their clinical application is hampered by technical difficulties^11,12^.

Once the model has been defined, comprehending the complex nature of the tumour microenvironment is essential in order to gain an in-depth knowledge of cancer biology at a single-cell level^13^. Current high-resolution imaging systems can be used to understand cell-cell interactions within spheroids. For instance, light-sheet fluorescence microscopes (LSFMs) provide better imaging depth, faster imaging, less phototoxicity, and less photobleaching compared to wide-field or confocal microscopes. Furthermore, the optical sectioning results in less light-scattering and provides high-resolution plane-by-plane imaging, allowing the visualisation of single cells even in a dense 3D microenvironment^14^.

In this work, we created 3D tumour multiculture spheroids composed of three different fluorescently labelled cell lines analysed at different time points. In particular, we created three different tumour models composed of one cancer cell line (*i.e*., melanoma, breast cancer, or osteosarcoma), labelled with CellTracker (CT) Orange CMTMR dye, and two stromal cell lines, *i.e*., fibroblasts and endothelial cells, stained with CT DeepRed and CT Green CMFDA, respectively. To mimic chemotherapeutic stress, we applied low-dose doxorubicin (dox), which is a widely used antineoplastic drug in a myriad of malignancies^15^. Then, using the Leica True Confocal Scanning (TCS) SP8 Digital LightSheet (DLS) microscope, we acquired single-cell light-sheet fluorescence 3D images of the generated spheroids to analyse the cellular distribution after 24, 48, and 96 hours. While tracking structural changes in spheroids over an extended period can be informative, the high level of cell-to-cell communication led to the exchange of cytoplasmic dyes between different cell types, making it challenging to distinguish them. Finally, an expert microscope operator performed the segmentation of the entire spheroids and extracted morphological features using publicly available tools, *i.e. AnaSP*^16,17^ and *ReViSP*^18^.

The acquired LSFM 3D images of multiculture spheroids, the obtained segmentation masks and the extracted morphological features are freely available at figshare^19^. They can be used for further biological investigations on the cell distribution in 3D environments, for instance to investigate the position of the cancer cells over time when co-cultured with healthy stromal cells^20^. In addition, the 3D data can be exploited by the research community to compare various computational metrics quantitatively to assess image quality^21^, or to generate training sets for deep learning and machine learning techniques^22^, as well as to validate other segmentation approaches^23^.

## Methods

### Cell lines description

In this work, we used five different commercial human cell lines obtained from the American Type Culture Collection (ATCC, Manassas, Virginia, USA); three cancer cell lines*, i.e*. T-47D ductal carcinoma (ATCC, HTB-133, Lot: 63542749), A375 melanoma (ATCC, CRL-1619, Lot: 63905420), MG-63 osteosarcoma (ATCC, CRL1427, Lot: 70054704), and two stromal cell lines, *i.e*. MRC-5 fibroblasts (ATCC, CCL-171, Lot: 63405646), and EA.hy926 endothelial cells (ATCC, CRL-2922, Lot: 70030244). They were maintained following the corresponding ATCC guidelines.

### Spheroid generation and staining

Multiculture tumour spheroids were generated by co-culturing three cell lines, specifically MRC-5, EA.hy926, and one type of tumour cell, either T-47D, A375, or MG-63. They were co-cultured in 384-well ultra-low attachment U-bottom plates (Greiner Bio-One, Kremsmünster, Austria), in DMEM supplemented with 10% Opticlone FBS (EuroClone, Milan, Italy), 2 mM L-glutamine and 1% Penicillin-Streptomycin-Amphotericin B mixture (all from Lonza, Basel, Switzerland). Before seeding, cells were stained with CellTracker dyes based on the manufacturer’s instructions (Invitrogen, Thermo Fisher Scientific, Waltham, Massachusetts, USA). Precisely, MRC-5, EA.hy926, and the tumour cells were stained with 1 µM Deep Red, 25 µM Green CMFDA, and 25 µM Orange CMTMR dyes, respectively. Then, the cells were mixed to generate tri-culture spheroids; we used 40% MRC-5, 40% EA.hy926, and 20% tumour cells. Spheroids were incubated for 24, 48, or 96 hours at 37 °C and 5% CO_2_ in the control medium or 0.6 µM dox-containing medium. Before live-cell imaging nuclei were stained with 1.5 µM Hoechst 33342 in DMEM for 60 min.

### Image acquisition

The imaging setup and all imaging parameters were previously described in *Diosdi et al*.^24,25^. Briefly, for each spheroid, fluorescent 16-bit images were produced using a Leica TCS SP8 DLS microscope with a sCMOS DFC9000 camera (Leica Microsystems, Wetzlar, Germany). The images were acquired at three different time points (*i.e*., 24, 48, and 96 hours after seeding) using a 200 ms exposure time and a 25x/0.95 detection objective with a 2.5 mm mirror device on the objective. The laser intensity was adjusted for each channel at 405, 488, 552, and 638 nm (maximum laser intensity 350 mW). For imaging, DPBS mounting medium was used for every spheroid. The resolution of the images is 2048 × 2048 pixels, with a pixel size of 0.14370117 µm and a distance of 3.7 µm between each image in z-stack. The Leica Application Suite X (LAS X) software produced Maximum Intensity Projection (MIP) 8-bit images with a pixel resolution of 1683 × 1683 and a pixel size of 0.17486631 µm.

### Image analysis

Morphological features have been extracted by analysing the MIP of each spheroid reported in the collection using *AnaSP* version 3.0^17^. Briefly, MIP images were obtained by merging all the fluorescent channels and these were manually segmented by an expert microscope operator for obtaining binary masks, with the foreground in white over a black background. Several morphological features (*e.g*., *Diameter*, *Perimeter*, *Area*, *Circularity*, and *Sphericity*) have been extracted, including the *Volume*, which was reconstructed using *ReViSP*. The mathematical equations describing the features are available in the *AnaSP*’s user manual and Supplementary File 1.

## Data Records

**Fig. 1:**
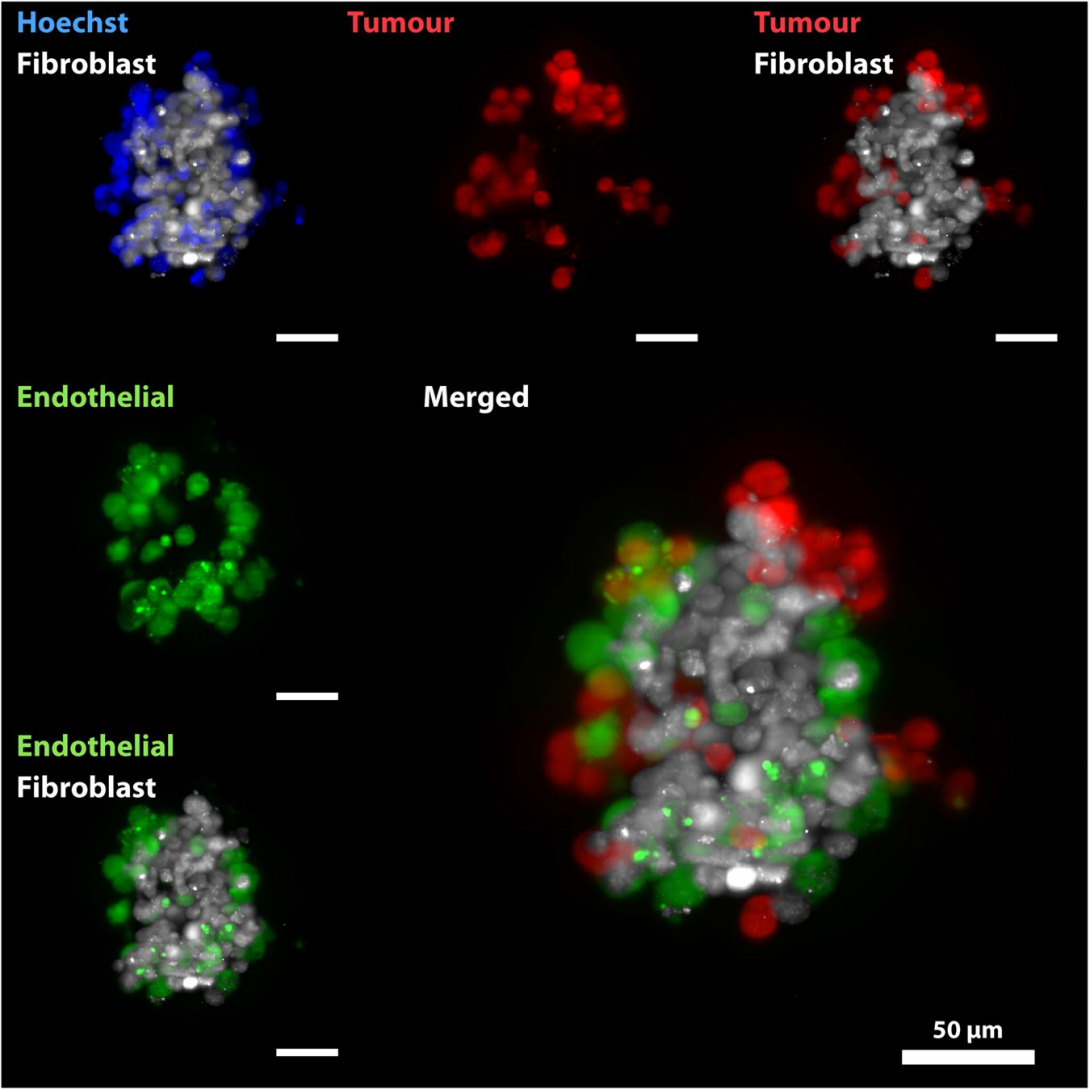
Representative Maximum Intensity Projection (MIP) images of a 3D tumour multiculture spheroid of the A375 cell line imaged 24 hours after seeding. Four channels were acquired for visualisation, precisely Blue - Nucleus (Hoechst 33342), White - MRC-5 (Deep Red), Green - EA.hy926 (Green CMFDA), and Red – A375 (Orange CMTMR). The scale bar represents 50 µm for all the images. Images were taken and visualised using Leica LAS X software.

The dataset is available at figshare^19^, with this section being the primary source of information on the availability and content of the data being described. The collection includes images of spheroids acquired at three time points from the generation of the multiculture 3D tumour models. The images are single-cell light-sheet fluorescence 3D data saved in a multi-tiff file format. Each spheroid was stained with the same four fluorescent dyes. For each staining, a 3D stack is provided for each spheroid. Figure 1 reports the MIP for the four channels (Nuclei - ch001, EA.hy926 - ch002, tumour - ch003, and MRC-5 - ch004) available in the collection for a random spheroid shown as an example. All spheroids are acquired from top to down and each layer of the tiff file corresponds to a different z-section. The number of layers ranges from 38 to 90 sections, and the z-step between subsequent layers is the same for all the spheroids. In a separate folder MIP images are also provided for each spheroid and all of the four channels and a merged one.

**Fig. 2:**
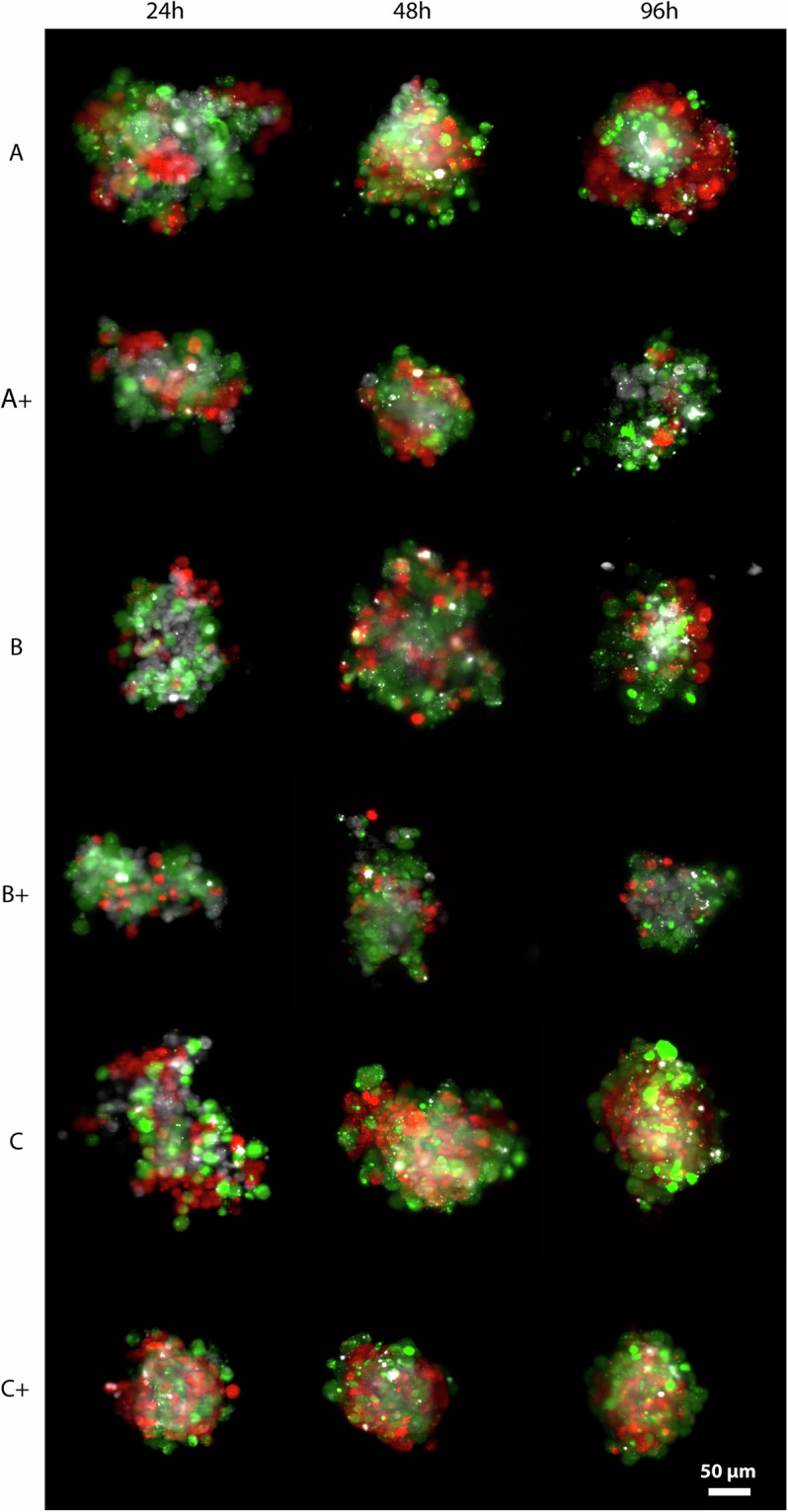
Representative Maximum Intensity Projection (MIP) images of the 3D tumour multiculture spheroid dataset. The three different tumour types were labelled as A - T-47D, B - A375, and C - MG-63, and dox- treated samples were differentiated with “+” symbol. For visualisation, three channels were merged, precisely White - MRC-5 (Deep Red), Green - EA.hy926 (Green CMFDA), and Red - Tumour (Orange CMTMR dyes). The scale bar represents 50 µm. The images were taken and visualised using Leica LAS X software.

For each tumour cell line and time point, images of 5 spheroids have been acquired for both the control and the drug-treated condition. The three different tumour types were labelled as A - T-47D, B - A375, and C - MG-63, and dox-treated samples were differentiated with “+” symbol. In total, 90 spheroids are included in the collection according to the scheme reported in Table 1. All the data are freely available at figshare^19^. The uploaded files follow the naming convention: “TumourType_ + _TimePoint_SpheroidID_channelID.tif” where (+) labels doxorubicin treated samples. Figure 2 shows a random spheroid for each condition obtained using the different fluorescence channels, excluding the nuclei signal for a better understanding of the cell distribution.

**Table 1:** Collection statistics: number (n.) of spheroids and layers for each condition considered.

Multiculture spheroids	24 h		48 h		96 h		Total	
	N. of spheroids	Total n. of z-layers	N. of spheroids	Total n. of z-layers	N. of spheroids	Total n. of z-layers	N. of spheroids	N. of z-layers
T-47D (A)	5	285	5	309	5	315	15	909
dox-T-47D (A+)	5	266	5	259	5	250	15	775
A375 (B)	5	284	5	286	5	269	15	839
dox-A375 (B+)	5	280	5	319	5	242	15	841
MG-63 (C)	5	293	5	281	5	287	15	861
dox-MG-63 (C+)	5	236	5	270	5	264	15	770
Total n.	30	1644	30	1724	30	1627	90	4995

For each spheroid included in the collection, several morphological features have been extracted using merged MIP images. The MIP images and related binary masks are freely available at figshare^19^. For each spheroid analysed, Supplementary Table 1 reports values for selected features, Also, a table reporting all the values for the different extracted features is available in a .csv and .xls format at figshare^19^. The average values are reported in Table 2. The single values are reported in µm in the case of *Diameter* and *Perimeter*, in µm^2^ for *Area*, in µm^3^ for *Volume*, and dimensionless (*i.e*. [−]) for *Circularity* and *Sphericity*.

**Table 2:** Average values of selected morphological features for each group of five spheroids considered for each condition.

Time point	Multiculture spheroids	Diameter [µm]	Perimeter [µm]	Area [µm^2^]	Volume [µm^3^]	Circularity [−]	Sphericity [−]
24 h	T-47D (A)	174	713	24376	2574743	0.85	0.77
48 h	T-47D (A)	162	734	20564	1733780	0.76	0.69
96 h	T-47D (A)	167	680	21842	2218002	0.83	0.77
24 h	dox-T-47D (A+)	174	815	24412	2146463	0.74	0.68
48 h	dox-T-47D (A+)	161	673	20818	1830226	0.79	0.76
96 h	dox-T-47D (A+)	141	604	15758	1034803	0.76	0.74
24 h	A375 (B)	185	882	24376	2328160	0.72	0.66
48 h	A375 (B)	187	827	20564	2834749	0.78	0.72
96 h	A375 (B)	161	689	21842	1932300	0.79	0.74
24 h	dox-A375 (B+)	147	728	16989	1005072	0.69	0.64
48 h	dox-A375 (B+)	146	679	16828	1210798	0.73	0.68
96 h	dox-A375 (B+)	137	610	15004	1143153	0.75	0.72
24 h	MG-63 (C)	171	791	27817	1873964	0.78	0.71
48 h	MG-63 (C)	178	767	27848	1989199	0.79	0.73
96 h	MG-63 (C)	178	730	20451	2523054	0.82	0.77
24 h	dox-MG-63 (C+)	146	631	16991	1408364	0.81	0.75
48 h	dox-MG-63 (C+)	143	583	16171	1315481	0.81	0.78
96 h	dox-MG-63 (C+)	140	556	15461	1220975	0.83	0.79

## Technical Validation

All images were collected as part of routine wet-lab analyses using a commercial microscope. Accordingly, quality assurance was performed just with daily calibrations done on the system. The raw images were uploaded without editing to figshare. Two expert microscopists carefully checked the uploaded data in blind.

## Supplementary information

Supplementary File 1.

Supplementary Table 1.

## Data Availability

For spheroid feature analysis the open-source tools *AnaSP*^16,17^ and *ReViSP*^18^ were used. Custom codes have not been designed and used for this study.
