# Supplementary material for: Single-cell light-sheet fluorescence 3D images of tumour-stroma spheroid multicultures
Source: Sci Data. 2025 Mar 24;12:492. doi: 10.1038/s41597-025-04832-0 (PMC11933373; doi:10.1038/s41597-025-04832-0)
Supplement: Supplementary file 1 [file 41597_2025_4832_MOESM1_ESM.pdf]

LIST OF FEATURES:

1. "Area" = number of pixels of the foreground.

2. "Circularity = 4*pi*Area/(ConvexPerimeter^2)", with "Area=area of the original mask" and "ConvexPerimeter=perimeter of the convex mask".

3. "Compactness = 4*pi*Area/(Perimeter^2)", with "Area=area of the original mask" and "Perimeter=perimeter of the original mask", defined according to: Cox, E. P. (1927). A method of assigning numerical and percentage values to the degree of roundness of sand grains. Journal of paleontology, 1(3), 179-183.

4. "Convexity = ConvexPerimeter/Perimeter", with "ConvexPerimeter=perimeter of the convex mask" and "Perimeter=perimeter of the original mask".

5. "EquivalentDiameter" = diameter of a circle with the same area as the foreground, computed as "sqrt(4*Area/pi)".

6. "FeretAspectRatio" = ration between the "FeretDiameterMax" and the "FeretDiameterMax".

7. "FeretMaxDiameter" = maximum possible diameter masurable with a standard caliber used in the same x-y plane of the mask. See the first 5 figures of this blog to understand better: *https://blogs.mathworks.com/steve/2018/04/17/feret-properties-wrapping-up/*

8. "FeretMaxDiameterOrthogonalDistance" = length of the Orthogonal axis of the box related to the maximum possible diameter measurable with a standard caliber used in the same x-y plane of the mask. See the first 5 figures of this blog to understand better: *https://blogs.mathworks.com/steve/2018/04/17/feret-properties-wrapping-up/*. Please, note that the angle between the "FeretDiameterMax" and the "FeretDiameterMin" is not 90° most of the times, while the angle between the "FeretDiameterMax" and the "FeretDiameterMaxOrthogonalDistance" is always 90°.

9. "FeretMinDiameter" = minimum possible diameter masurable with a standard caliber used in the same x-y plane of the mask. See the first 5 figures of this blog to understand better: *https://blogs.mathworks.com/steve/2018/04/17/feret-properties-wrapping-up/.* Please, note that the angle between the "FeretDiameterMax" and the "FeretDiameterMin" is not 90° most of the times, while the angle between the "FeretDiameterMax" and the "FeretDiameterMaxOrthogonalDistance" is always 90°.

10. "MaxDiameterThroughCentroid" = maximum axis passing through the centroid of the foreground.

11. "MinDiameterThroughCentroid" = minimum axis passing through the centroid of the foreground.

12. "Perimeter" = number of pixels of the perimeter of the foreground.

13. "Solidity = Area/ConvexArea", with "Area=area of the original mask" and "ConvexArea=area of the convex mask".

14. "Sphericity = pi*sqrt(4*Area/pi)/Perimeter", with "Area=area of the original mask" and "Perimeter=perimeter of the original mask", defined according to: Kelm, J. M., Timmins, N. E., Brown, C. J., Fussenegger, M., & Nielsen, L. K. (2003). Method for generation of homogeneous multicellular tumor spheroids applicable to a wide variety of cell types. Biotechnology and bioengineering, 83(2), 173-180.

15. "Volume" = number of voxels of the volume of the foreground, reconstructed using the ReViSP [*Piccinini et al., Cancer multicellular spheroids: Volume assessment from a single 2D projection. CMPB, 118(2):95–106, 2015*] algorithm.
